# Parental Experiences in Pediatric Multiple Sclerosis: Insights from Quantitative Research

**DOI:** 10.3390/children11010071

**Published:** 2024-01-08

**Authors:** Samuela Tarantino, Martina Proietti Checchi, Laura Papetti, Gabriele Monte, Michela Ada Noris Ferilli, Massimiliano Valeriani

**Affiliations:** 1 Developmental Neurology Unit, Bambino Gesù Children’s Hospital, Istituto di Ricovero e Cura a Carattere Scientifico (IRCCS), 00165 Rome, Italylaura.papetti@opbg.net (L.P.); massimiliano.valeriani@opbg.net (M.V.); 2 Systems Medicine Department, Tor Vergata University of Rome, 00133 Rome, Italy; 3 Center for Sensory-Motor Interaction, Aalborg University, 9220 Aalborg, Denmark

**Keywords:** multiple sclerosis, children, adolescents, family, parental stress, parental burden

## Abstract

Multiple sclerosis (MS) is a chronic and unpredictable inflammatory disease impacting the central nervous system. The disabling nature of this disease is not limited to only physical symptoms. MS, even at a pediatric age, often includes cognitive impairment, fatigue, and psychological issues, affecting education and social life, causing emotional distress, and reducing quality of life. Despite the paucity of quantitative data in the existing literature, our review demonstrates that the impact of pediatric MS extends beyond the patients themselves, affecting their parents as well. There is evidence suggesting that having a child with MS may be associated with a reduction in the parental quality of life, even in families of MS patients with low or no disability and without clinical relapses. Moreover, an increased risk of parents’ mental illness has been described, particularly in mothers, leading to a heightened utilization of mental health services. Research data show that inadequate information about MS may impact parents’ anxiety and their sense of competence. Since parents’ involvement has been found to also play a role in their child’s adherence to treatment, special attention should be paid to parental psychological health. Additional research exploring family adaptation to their children’s illness is required.

## 1. Introduction

Multiple sclerosis (MS) is a chronic and unpredictable inflammatory disease impacting the central nervous system [1,2]. It presents with brain and spinal cord lesions that can result in physical and cognitive impairments [3,4]. While typically diagnosed in adults (adult-onset MS or AOMS), pediatric-onset multiple sclerosis (POMS), referring to the manifestation of MS in individuals under 18 years old, involves from 3% to 10% of the entire MS population [5,6,7]. The clinical presentation of POMS varies with age [8]. Younger children under 11 often have multiple symptoms, while those over 12, like adults, typically present with a single symptom [9]. Over 90% of POMS cases are diagnosed as relapsing–remitting, with more frequent early relapses, indicating high inflammation [10]. Children have better recovery due to their developing nervous tissue and higher myelin repair but reach significant disability earlier, typically in their 30s [11,12].

MS leads to different symptoms, such as sensory disturbances, mobility issues, and vision problems [2,7]. MS, even at a pediatric age, often includes cognitive impairment, fatigue, and psychological issues, affecting education and social life, causing emotional distress, and reducing quality of life [13,14,15]. Patients often experience feelings of hopelessness, possibly linked to organic factors like reduced brain activity [16]. The uncertainty for the future, particularly when MS occurs early in life, threatens many dreams and projects typical of adolescence, such as independence, travel, relationships, personal growth, education, and career success [17].

Receiving a diagnosis of a chronic illness can also have a profound and enduring impact on the entire family, particularly when the condition is relatively uncommon, as in case of POMS [18]. Literature data indicate that parents of chronically ill children grapple with emotional distress and vulnerability [19]. Qualitative data suggest that families receiving a POMS diagnosis undergo a complex process of change, acceptance, and adaptation to the disease [17,18,20,21]. From the onset of symptoms, patients and parents may endure heightened levels of anxiety and stress, exacerbated by months of uncertainty before a conclusive diagnosis is reached [20,21,22]. Given the unpredictable nature of the MS course, the process of accepting the final diagnosis may be accompanied by concerns regarding the potential for relapses or disease progression [17,20]. Analogous to parents of children with other chronic diseases, parents of children with MS may harbor concerns about how the illness might impede their children’s self-reliance, autonomy, future family planning, and overall quality of life [20,21]. Parents may experience feelings of worry and anxiety for their children and increased emotional, financial, and physical burdens associated with short- and long-term care [23,24,25,26].

Previous research on pediatric chronic patients indicate that parental stress significantly affects children’s illness management, potentially reducing treatment adherence, pain management, and overall well-being and resulting in increased symptoms and diminished quality of life for the child or adolescent [27,28,29]. 

While there is an extensive body of evidence highlighting the potential impact of AOMS on families, patients’ caregivers, and children [30,31,32,33,34], the experience of parents impacted by POMS remains inadequately investigated. In particular, although there is a growing focus on this subject, many studies in this area use a qualitative approach, which may inherently limit generalizability and not capture the full spectrum of experiences [17,18,20,21,22,26]. 

In this narrative review article, we aimed to focus on the burden of POMS on parents. Through an examination of the existing literature, with an emphasis on quantitative data, we aim to elucidate the parental burden in POMS, especially in terms of mental health, quality of life, and family relationships. Furthermore, to gain insights into patients’ adherence to therapy, we investigate existing studies concerning the role and participation of parents in the treatment of children with MS.

## 2. Methods

Relevant studies were identified through a comprehensive search of MEDLINE, Web of Science, Embase, and Scopus databases. Our research included studies published until October 2023. Our search was limited only to the English language. We used search terms such as “Pediatric Multiple Sclerosis” or “Pediatric Onset Multiple Sclerosis” in combination with keywords like “Family impact”, “Caregivers”, “Parental burden,” “Parental stress,” “Parental quality of life”, “Parental Mental illness”, “Parental involvement”, “Parental knowledge”, and “Family relationships”. Our search encompassed patients aged from 0 to 18 years, as well as articles involving adult patients diagnosed before the age of 18. We considered various types of studies, including observational, prospective, and retrospective studies, as well as clinical trials and multicenter studies. We specifically sought studies employing validated assessments for parental stress, quality of life, anxiety, and depression. 

In the initial study selection phase, two independent reviewers (S.T. and M.P.C.) evaluated the relevance of each study’s objectives based on their titles, abstracts, and keywords. Initial exclusion criteria involved duplicated articles. Subsequently, articles lacking reference to the psychosocial and familial impact of pediatric multiple sclerosis or those neglecting the burden on parents of affected children were excluded. In cases of consensus disparity among reviewers or insufficient abstract information, a comprehensive review of the complete text of the studies was conducted.

The second phase, which also included three other reviewers (L.P., G.M., and M.A.N.F.), involved a meticulous examination of the full texts to ascertain if they met the inclusion criteria. Any discrepancies or uncertainties during selection were addressed through consensus-building discussions, overseen by the senior author (M.V.). 

**Figure 1:**
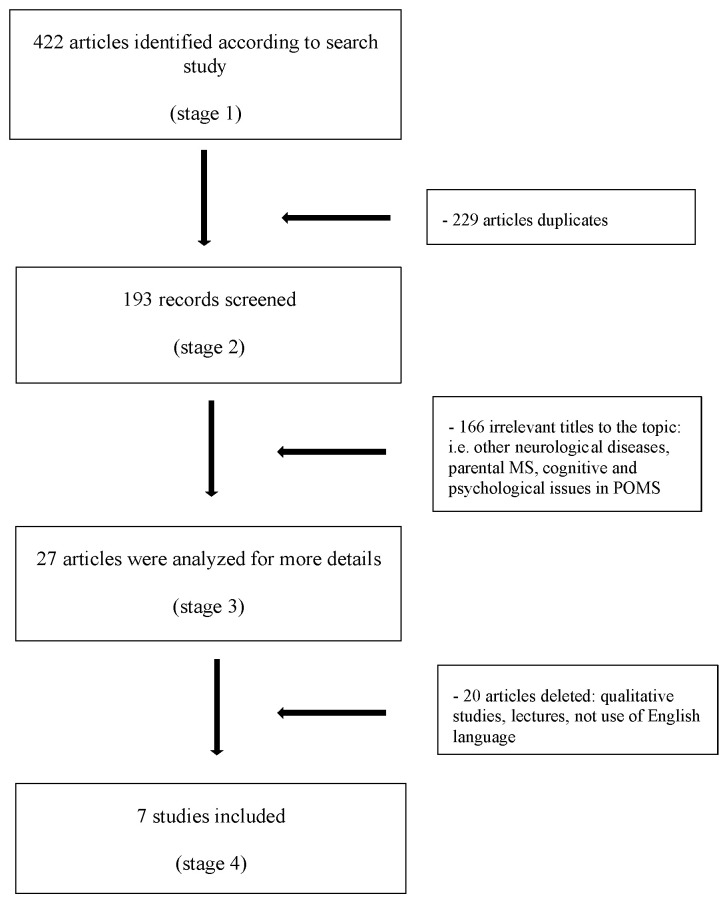
Flow diagram of the study methodology.

Following this, a thorough assessment was conducted on all studies concerning the condition’s impact on families and parents, excluding qualitative studies. The search specifically omitted non-full-length articles, poster presentations, and conference presentations (Figure 1).

## 3. Results

### 3.1. Parental Mental Health

Although very few empirical studies focused on parental mental health, there is evidence that having a child with MS may be associated with higher rate parental psychiatric symptoms. In 2013, Messmer Uccelli et al. described higher scores of depression in parents of children with MS (*n* = 15) as compared with those of parents of healthy children (*n* = 29) (Table 1) [24]. Parents of children with MS did not exhibit elevated anxiety levels compared to parents of healthy children, and no significant difference in anxiety was identified between mothers and fathers. Exploring parental worry, which refers to the emotional distress and concerns experienced by parents regarding the health, well-being, and safety of their children, the authors observed a significant discrepancy, with mothers displaying a markedly greater level of concern compared to fathers. Supporting previous qualitative studies, the authors supposed that although MS is categorized as a relapsing–remitting illness characterized by phases of stability, its inherent unpredictability can serve as a significant source of distress for mothers [24]. 

An increased burden of maternal mental illness has been described by Marrie et al., who investigated the prevalence of physical and mental conditions and the rate of healthcare utilization in mothers of children with multiple sclerosis (*n* = 156) (MS-mothers) (Table 1) [35]. MS-mothers exhibited a heightened occurrence of anxiety, mood disorders, and physical conditions before, during, and after their child’s diagnosis, as compared to mothers of children unaffected by MS (they had no hospital or physician claims for demyelinating disease). Furthermore, MS-mothers showed an increased tendency to utilize mental health services. Supporting the literature on chronic disease, the authors hypothesized that children’s MS diagnosis could result in an extended and stress-inducing experience for parents [19,35]. As an alternative explanation, the authors suggested that maternal mental illness might potentially heighten the susceptibility to pediatric MS [35].

### 3.2. Quality of Life

While the effects of MS on the quality of life of adult patients’ caregivers have been extensively studied [39,40], there is still a lack of comprehensive research addressing the distinct challenges and experiences faced by caregivers of pediatric patients with MS. Only in recent years, studies have started exploring the quality of life among the parents of pediatric patients diagnosed with MS. To the best of our knowledge, only four studies analyzed the impact of childhood MS on parent’s quality of life (QoL). In the aforementioned study by Messmer Uccelli et al., the authors assessed the quality of life of parents of pediatric patients with MS and compared it with that of healthy individuals [24]. The median quality of life score for parents of children with MS showed no significant differences compared to the control group. These findings led the authors to suggest that the impact of MS on parents is relatively milder compared to numerous other medical conditions. 

A negative influence of the disease on parental health-related quality of life (HRQoL) as well family functioning was described in a quantitative study by O’Mahony et al. (Table 1) [28]. The authors measured HRQoL in parents of individuals affected by MS (*n* = 58) and in those of patients with monophasic acquired demyelinating syndrome (monoADS, *n* = 178). Compared with parents of children with monoADS, parents of children with MS described reduced HRQoL in every area of the PedsQL-Family Impact Module, including parental cognitive, emotional, social, and physical functioning, parental worry and communication, family’s relationships, and daily activities [28]. Parental HRQoL was reduced even in families of MS patients with low or no disability and without clinical relapses. It was suggested that the discrepancies in HRQoL between parents of children with MS and monoADS are more likely to be influenced by the diagnosis of a chronic illness, rather than the activity or severity of the disease. A few years later, the same authors demonstrated that parental HRQoL could act as an intermediary factor in the correlation between the diagnosis of MS and the HRQoL of the affected children [25]. Particularly, the diagnosis of MS (in comparison to monoADS) was linked to decreased HRQoL among parents, which subsequently led to a decrease in HRQoL of the affected children (Table 1) [25]. 

In a recent study involving pediatric patients with MS (*n* = 65) and monoADS (*n* = 142), O’Mahony et al. found an additive effect of familiar health condition and low socioeconomic position on the association between pediatric MS and parental HRQoL [36]. Particularly, parents with a child diagnosed with MS were notably prone to experiencing reduced HRQoL in presence of either of these supplementary risk factors. Although the number of health conditions or low socioeconomic position showed no significant variation between families of children with MS and those with monoADS, clear differences emerged in how these factors influenced HRQoL (Table 1) [36].

### 3.3. Family Relationships

Difficulties within family relationships, conflicts, and a lack of unity have been linked to unfavorable health outcomes within families dealing with a child suffering from a chronic illness [41]. In the context of POMS, few studies, most of which adopting a qualitative method, have investigated the familial climate and the implications of the illness on the dynamics of couples. To the best of our knowledge, only one quantitative study explored family and couple relationships among families with POMS. In 2013, by using F-Copes test, a self-report assessment designed to identify effective problem-solving and behavioral strategies used by families in difficult or problematic situations, Messmer Uccelli et al. showed minimal distinctions between parent groups (POMS patients’ parents vs. healthy children’s parents), with the exception of the coping behavior [24]. This outcome led the authors to suppose that parents of children with MS demonstrate notable adaptability to their child’s medical condition. Moreover, the study did not find any significant difference between the groups in terms of the quality of the partner relationship. The authors suggested that the strength of the couple’s bond remains intact despite the diagnosis of MS in the child [24]. The notion of parenting sense of competence encompasses both an individual’s perceived self-efficacy as a parent and the contentment they derive from their parental role [42,43]. Although the above-mentioned study did not identify significant differences in parenting efficacy or satisfaction between mothers and fathers of children with MS, parents in the MS group reported notably lower levels of satisfaction with their parenting role when compared to control parents [24]. These results could be due to the unpredictable nature of MS, which could limit parental control and proactive care, leading to reduced satisfaction in parenting role [24]. Additionally, the “invisible” symptoms of MS may have contributed to parental ambivalence about their role and identity, potentially impacting satisfaction. A significant statistical correlation was observed between limited knowledge about the child’s illness and the overall sense of parental competence. Consequently, inadequate information about MS can significantly impact family dynamics, elevate parental anxiety, and diminish their perception of competence [24]. In line with previous qualitative research, the authors demonstrated that limited knowledge of MS significantly correlated with decreased couple relationship satisfaction and communication quality [17,24].

### 3.4. Parental Involvement and Satisfaction with Treatment

The complexity of multiple sclerosis treatment plans, often including disease-modifying therapies (DMTs) administered through injections, infusions, or oral medications, together with emotional and social factors, can be a significant factor contributing to non-adherence [44,45,46]. Although disease-modifying therapies (DMTs), such as fingolimod, natalizumab, and rituximab, may lead to better disease control and a reduced relapse rate in pediatric MS, the use of chronic medication may be troublesome [46]. Parents may express frustration due to the uncertain benefits of DMTs and the challenges associated with injections, particularly with agents like interferon-beta, a common first-line treatment, known to frequently cause minor side effects such as injection site swelling and flu-like symptoms [17,20,46]. Choosing a DMT for a child with a new diagnosis of POMS can represent a significant challenge for many parents [20,38,47]. So far, there is a scarcity of quantitative studies on the role and involvement of parents of patients with MS in their children’s therapy. In 2018, Schwartz et al. identified a relationship between parental involvement in their child’s administration of DMT and the child’s adherence to DMT [37]. While the type of DMT did not impact adherence, differences in parental involvement were observed based on the mode of administration, with less involvement in oral medications compared to injectable ones. Moreover, the study showed that parental involvement in adherence could play a crucial protective role in pediatric MS, especially for adolescents who encounter difficulties in school and exhibit lower confidence in managing their disease (Table 1) [37].

It has been shown that parental satisfaction may be closely linked to their involvement in the decision-making process, with their level of contentment regarding the chosen DMT being directly correlated to their sense of control and participation in that decision [37]. In a study conducted on 97 parents of children and adolescents diagnosed with MS, Duffy et al. explored the decision-making experience among parents of children who have received or were receiving medication for their MS [38]. The authors evidenced that less than half of parents of their sample (44%) expressed a high level of satisfaction with the DMT they selected for their child. Parental satisfaction was positively associated with having a high level of control over the process, being content with communication, and feeling supported by the healthcare provider (Table 1) [38].

## 4. Discussion

Pediatric multiple sclerosis is a progressive disease characterized by an unpredictable course and adverse effects on patients’ physical, cognitive, and psychological well-being [1,2,13,28,48,49]. Despite the paucity of quantitative data in the existing literature, our review demonstrates that the impact of pediatric MS extends beyond the patients themselves, affecting their parents as well [24,25,28,35,36,37,38]. The diagnostic processes and the subsequent communication of the diagnosis can be overwhelming for both parents and patients, triggering a range of emotional responses, including feelings of shock and worry [20,21,22,24]. When a child is diagnosed with a chronic illness like MS, parents often take on the role of caregivers, necessitating significant adjustments in their daily lives [17,21,50] The caregiving dynamics can differ significantly for either pediatric or adult MS patients [22,50]. While the deep attachment between parent and child remains, the additional responsibilities and potential lifestyle changes can be overwhelming, leading to a substantial emotional and psychological burden [24,26,28,35,50]. Parents of pediatric patients with a chronic disease must balance typical parenting duties with managing medical needs, appointments, treatments, and potential lifestyle adjustments required by the condition. They manage medical decisions, provide emotional support, advocate for their child’s needs within the healthcare system, and often manage daily tasks related to the disease [51]. Due to the profound emotional burden they carry, in the literature, these parents are often referred to as “hidden patients”, not immediately visible to healthcare professionals [52,53,54]. This combination of stressors can negatively affect the overall quality of life of POMS patients’ parents [24,25,28,36]. Extensive research consistently highlights a notable reduction in the quality of life among parents who become primary caregivers for pediatric patients with chronic conditions [51,55,56,57,58]. Available data of the literature indicate that the impact of MS on parental quality of life is primarily attributed to the chronic nature of the disease itself, rather than its specific symptomatic manifestations [17,24,28]. This influence extends across various domains, encompassing cognitive, emotional, social, and physical functioning, in addition to its effects on family relationships and daily activities [13,14,17,24,26,48]. Emphasizing the critical role of parental well-being, research data have demonstrated that the HRQoL of parents can serve as a mediating factor in the correlation between the diagnosis of MS and the HRQoL of the affected children [36].

As emerged in qualitative research, one significant factor contributing to the challenges faced by parents and caregivers is the unpredictable nature of pediatric MS [17,21,24]. The constant worry about disease progression, treatment decisions, and long-term outcomes can contribute to high levels of stress and anxiety among parents, especially in mothers [24]. This unpredictability, coupled with a lack of control over their child’s condition, can lead to decreased satisfaction in their parenting roles [24]. Another contributing factor to the parental burden in POMS is the lack of knowledge or awareness about the condition. The reviewed literature highlights that limited knowledge about child’s illness, specifically MS, is statistically linked to lower parental competence and affects family dynamics negatively [24]. It also mentions that inadequate MS information can lead to increased parental anxiety and strained couple relationships. Conversely, parental satisfaction is positively correlated with factors such as control, effective communication, and support from healthcare providers [38].

These findings underscore the need for emotional support for parents regardless of their child’s disease status [28]. Given the influence of parental quality of life on their children’s well-being, it is crucial to delineate methodologies and factors contributing to mitigating anxiety and stress levels in parents of children and adolescents diagnosed with POMS [36]. However, to the best of our knowledge, no quantitative study explored stress management methods in parents of patients with POMS. The available insight on this field primarily comes from qualitative studies [21,22,26,59]. Parents of children with POMS highlighted critical themes, such as enhancing medical community awareness, parental involvement in diagnosis disclosure, active listening, and comprehensive family resources [17,18,20,21]. Healthcare providers may play a crucial role in guiding families through initial diagnosis discussions, tailoring communication based on the child’s age and preferences, and ensuring careful planning and preparation for these discussions. Krupp et al. suggested that the optimal approach for sharing the diagnosis, as well as discussing disease management, may differ depending on the child’s age [60]. Maintaining balance between the needs of parents and patients can be challenging, underscoring the importance of psychological support for the entire family during the diagnostic process [18,20]. Supporting the parental need for appropriate information could be very helpful in order to deal with their own emotions and determine how to help their child [17,21]. Assisting parents in understanding signs of relapse and knowing when to seek medical help may empower parents, providing them with a greater sense of control over their child’s illness [21]. What was previously uncertain and frightening could have the potential to become “known” and therefore manageable. From this point of view, specialists, general practitioners, communities service, friends, and family may all become key sources of knowledge and support [17,20]. 

Despite the extensive research on psychological support for caregivers of adult MS patients, the research landscape notably lacks studies focusing on tailored psychotherapeutic approaches for pediatric MS and parents of children with POMS. Cognitive–Behavioral therapy (CBT), known for effectively managing depression and fatigue in adult MS [61,62], can target issues such as fatigue perceptions and emotional and behavioral patterns in both parents and children affected by MS [22]. 

The main limitation of this review article is that it is narrative in nature. This poses a problem regarding the generalizability of the results. Unfortunately, the number of quantitative studies addressing psychological burden in parents of children with POMS is too low for a systematic review. Despite this limitation, we believe that a review of the available literature is important for two reasons: (1) it sheds light on an important topic that is poorly represented in the scientific literature and (2) it can provide a backdrop for future studies.

## 5. Conclusions

Pediatric MS is very challenging for parents, affecting multiple aspects of their lives. Recognizing the profound significance of parental health and well-being, both emotional and physical, in shaping the course of their children’s illness, understanding and addressing these intricate and multifaceted challenges are crucial for healthcare professionals. By offering comprehensive support, personalized interventions, and readily accessible resources, we can facilitate adaptive coping mechanisms and enhance the overall quality of life for both young patients and their caregivers. Furthermore, recognizing the unique challenges faced by caregivers of pediatric MS patients and conducting further research in this area will contribute to better interventions and ultimately enhance the overall well-being of both caregivers and their young children.

## Figures and Tables

**Table 1:** Summary of the results obtained from the selected studies.

Author	Study Design	Sample	Aspects Assessed	Assessment Tools	Main Results
Messmer Uccelli et al., 2013 [24]	Cross-sectional observational study	15 couples with a child with MS and 29 couples with healthy children Diagnosis of MS ≤16 years of age	How couples raising a child with MS handle family crises and individual distress in comparison to couples with healthy children	- MWS - Four ENRICH Couple scales - F-COPES - PSOC - HADS - WHO-Five Well-being Index - MSKQ	Parents of children with MS felt less confident and satisfied in their parenting compared to control parents. Even though they showed higher parental depression levels than the control group, these scores were still within the normal range. Limited MS knowledge was associated with poorer relationship satisfaction and communication quality, impacting overall parental competence
Marrie et al., 2020 [35]	Population-based, retrospective matched cohort study	156 mothers of children with MS (MS-mothers) and 624 mothers of children with no hospital or physical claims for demyelinating diseases (non-MS-mothers) Diagnosis of MS ≤18 years of age	The occurrence of physical and mental health conditions and the frequency of healthcare utilization in MS-mothers in comparison to non-MS-mothers	None	MS-mothers tended to utilize mental health services more frequently both before and after their child’s MS diagnosis compared to non-MS-mothers
O’ Mahony et al., 2019 [28]	Prospective cohort study	58 MS and 178 monoADS patients and their parents Patients ≤ 16 years of age	The impact of chronic illness on children diagnosed with MS and their families in contrast to those with monoADS, examining their health-related quality of life and their perception of family functioning	- PedsQL child report - PedsQL parent’s self-report Family Impact Modules module Multidimensional Fatigue module	Parents of children with MS experienced lower HRQoL and poorer family functioning compared to parents of children with monoADS. Parents of MS-affected children reported more emotional dysfunction, increased worry, poorer communication, and reduced family functioning, regardless of clinical disease activity
O’ Mahony et al., 2022 [25]	Prospective cohort study	65 MS and 142 monoADS parent–child dyads Patients aged <18 years	The role of parental HRQoL as a mediator between the MS diagnosis and the HRQoL of affected children	- PedsQL child report - PedsQL parent self-report Family Impact Modules	Parental HRQoL played a mediating role between the MS diagnosis and the HRQoL of affected children
O’ Mahony et al., 2023 [36]	Prospective cohort study	65 MS and 142 monoADS parent–child dyads Patients aged <18 years	The potential roles of a family health condition and low socioeconomic position on the relationship between an MS diagnosis in childhood and parental HRQoL	- PedsQL child report - PedsQL parent self-report Family Impact Modules - BSMSS	Parents of children with MS, facing another family health condition or a low socioeconomic position, were at a heightened risk for experiencing low HRQoL
Schwartz et al., 2018 [37]	Cross-sectional study	66 youth with MS and 66 parents Patients’ age at enrollment between 10 and 18 years	The prevalence and risk factors for poor adherence in pediatric MS	- MMAS child report - MMAS parent report - MSTAQ - PedsQL child report - PedsQL parents’ report - MSSE patients’ report - PWB patients’ report	Parental involvement in administering therapies was linked to better treatment adherence in pediatric MS, particularly beneficial for adolescents struggling in school and feeling less confident in managing their condition
Duffy et al., 2021 [38]	Cross-sectional study	97 parents of children and adolescents with MS	The decision-making experience among parents of children who have received or were receiving medication for their MS	Survey items	Parental satisfaction was positively associated with having a high level of control over the process, being content with communication, and feeling supported by the healthcare provider

MS, multiple sclerosis; MWS, Maternal Worry Scale; F-COPES, Family Crisis Oriented Personal Evaluation scales; PSOC, Parenting Sense of Competence; HADS, Hospital Anxiety and Depression Scale; MSKQ, Multiple Sclerosis Knowledge Questionnaire; monoADS, monophasic acquired demyelinating syndromes; PedsQL, Pediatric Quality of Live Inventory; HRQoL, health-related quality of life; BSMSS, Barratt Simplified Measure of Social Status; MMAS, Morisky Adherence Measure; MSTAQ, Multiple Sclerosis Treatment Adherence Questionnaire; MSSE, Multiple Sclerosis Self-Efficacy Scale; and PWB, Ryff Scales of Psychological Well-Being.

## Data Availability

No new data were created or analyzed in this study. Data sharing is not applicable to this article.
